# Dysregulation of Glu-GABA and reduction of triglycerides contribute to valproic acid-induced autism model in zebrafish

**DOI:** 10.1016/j.jlr.2025.100911

**Published:** 2025-09-23

**Authors:** Qiwen Sun, Xinyi Huang, Han Long, Jianhua Guo, Ruilin Zhang, Daru Lu, Hongyan Yao, Keji Jiang, Yan Pi

**Affiliations:** 1 State Key Laboratory of Genetic Engineering, National Demonstration Center for Experimental Biology Education, School of Life Sciences, Fudan University, Shanghai, China; 2 Key Lab of Artificial Organs and Computational Medicine, Institute of Translational Medicine, Zhejiang Shuren University, Hangzhou, Zhejiang, China; 3 School of Basic Medical Sciences, Hubei Provincial Key Laboratory of Developmentally Originated Disease, Wuhan University, Wuhan, Hubei, China; 4 East China Sea Fisheries Research Institute, Chinese Academy of Fishery Sciences, Shanghai, Shanghai, China

**Keywords:** autism, metabolites, zebrafish, brain, lipid, neurotransmitter

## Abstract

Autism spectrum disorders are neurodevelopmental conditions that pose substantial diagnostic and therapeutic challenges. Maternal exposure to valproic acid (VPA) during pregnancy is a well-established risk factor associated with autism-like behaviors in offspring. This study characterized the metabolic phenotypes in the brain tissue of larval zebrafish following VPA exposure. Zebrafish were exposed to 4 μM VPA from 2 h postfertilization until 4.5 days postfertilization, and locomotor activity was assessed at 14 days postfertilization. Comprehensive metabolomic profiling via ultra-performance liquid chromatography-MS/MS identified 2,613 metabolites in brain tissue, of which 50 showed potential links to autism (CTRL_CV <15%, VPA_CV <20%). Significant reductions were observed in the levels of glutamine, glutamate, and triglyceride (TG). Nile red staining confirmed profoundly decreased TG deposition in the dorsal telencephalon (pallium), habenula, and cerebellum of VPA-exposed zebrafish. Furthermore, in vivo imaging revealed attenuated fluorescence intensity in excitatory glutamatergic and inhibitory gamma-aminobutyric acidergic neurons within the habenular nucleus and optic tectum, corresponding to reduced TG levels. Conversely, the cerebellar corpus (central cerebellar body) and inferior olive nucleus exhibited an increase in excitatory glutamatergic neurons and a reduction in inhibitory gamma-aminobutyric acidergic neurons, indicating an excitatory and inhibitory imbalance. Collectively, these findings suggest that VPA may promote autism pathogenesis by disrupting the glutamine-glutamate cycle and impairing TG metabolism in the zebrafish brain. These findings offer novel insights into metabolic dysfunction in autism spectrum disorders and may facilitate the identification of potential diagnostic biomarkers.

Autism spectrum disorder (ASD) is a neurodevelopmental disorder that manifests during early childhood and has enduring effects throughout an individual’s life (1). The etiology of ASD remains largely unidentified, which poses challenges for early diagnosis until distinct behavioral characteristics, such as difficulties in social interaction and the presence of repetitive behaviors, become apparent. ASD is characterized as a spectrum disorder due to the diverse range of symptoms and associated comorbidities, which may include aggression, hyperactivity, epilepsy, anxiety, sleep disturbances, attention-deficit/hyperactivity disorder, intellectual disabilities, and various mental disorders (2). The global prevalence of ASD is estimated to be approximately 1 in 100, with contributing factors that encompass genetic, biological, and environmental influences; over 1,000 risk genes have been identified in recent decades (3, 4). Current therapeutic approaches focus on alleviating symptoms that impact daily functioning, as there are no definitive curative treatments available currently.

Valproic acid (VPA) is a derivative of valeric acid, which is a short-chain FA and histone deacetylase inhibitor, and received approval in the 1980s for the treatment of epilepsy, bipolar disorder, and metabolic disorders, attributed to its anticonvulsant, neuroprotective, and neuroregenerative properties (5). However, the use of VPA during pregnancy has been consistently linked to the emergence of neurological disorders, including autistic-like behaviors and developmental disorders in offspring (6, 7, 8). VPA may work through the following mechanisms: inhibition of voltage-gated sodium channels (9), inhibition of gamma-aminobutyric acid (GABA) transaminase (10), enhancement of GABA synthesis (11), inhibition of histone deacetylases (12), and modulation of calcium channels (13). Nevertheless, the precise pathogenic mechanism remains incompletely understood. Experimental models utilizing zebrafish, mice, and nonhuman primates corroborate these associations, indicating that VPA exposure can induce disease models in zebrafish characterized by social defects (14).

Zebrafish serve as a remarkable model organism for investigating neurodevelopmental and degenerative diseases like Parkinson’s disease (15) and Alzheimer's disease (16). Several neurotoxins can be used to model neurodegeneration in zebrafish, including hydrogen peroxide (17), aluminum (18), aluminum chloride (19), bisphenol A (20), and some toxicity research (21). This capability enables researchers to simulate critical pathological mechanisms, such as oxidative stress, inflammation, and apoptosis. The versatility of zebrafish is further enhanced by the availability of diverse behavioral metrics that are relevant to neural function, such as locomotor velocity, immobility, shoaling, color preference, and responses to light-dark transitions. Behaviors associated with anxiety are also recognized as indicators of ASD behaviors in zebrafish (22). Thigmotaxis, which is posited to correlate with anxiety-like behavior in mammals (23), further establishes zebrafish as a relevant model for neurological disorders linked to locomotor dysfunction (24, 25).

Clinical investigations have revealed that individuals with ASD frequently exhibit abnormal metabolite profiles (3), thereby providing new avenues for the identification of autism biomarkers. Metabolomics, the high-throughput analysis of metabolites, emphasizes the profiling of the metabolic substrates and products within tissues, organs, or organisms (26). This methodology facilitates quantifying a wide array of low-molecular-weight metabolites, including amino acids and their derivatives, FAs, lipids, and organic acids, along with their derivatives (26), and provides an emerging tool for the exploration of disease mechanisms (27). Notably, lipids constitute approximately 50% of the brain's dry weight (28), and disruptions in lipid metabolism can precipitate various diseases. An increasing body of evidence underscores a direct relationship between metabolic processes and disease states, thereby positioning metabolomics as a crucial tool for investigating developmental and psychiatric disorders (26).

Existing research on the pathogenesis of autism and the mechanisms through which VPA induces autism is complex and fragmented. In this study, we employed VPA induction to treat zebrafish embryos from 2 h postfertilization to 4.5 days postfertilization (dpf), a crucial developmental window for neuronal tube formation (29), to investigate the mechanism by which VPA induces autism through metabolomics. Significant alterations in the glutamate cycle and triglyceride (TG) were identified in brain tissue metabolites of the VPA-induced zebrafish ASD model. This work lays a foundational understanding for further exploration of the connections between different brain regions and the mechanisms of autism treatment in the future.

## Materials and Methods

### Zebrafish maintenance

Wild-type zebrafish (*Danio rerio*, 8 months) used in this study were all AB line. They were kept in colonies in the zebrafish room following the 12 h light-12 h dark cycle (from 8 AM to 8 PM) with the constant 28°C water temperature in aerated water (pH: 7.2 ± 1.0). Zebrafish mating and embryo collection were conducted according to the protocol (30).

All experimental procedures were approved by the Institutional Animal Care and Use Committee of Fudan University. The ethical approval number was 2024JS060.

The Tg(*vglut2α:DsRed*) and Tg(*gad1b:DsRed*) lines were obtained from the National BioResource Project, Zebrafish, Core Institution (Saitama, Japan).

### Chemical treatment

The zebrafish model of autism was induced using VPA (CAS: 1069-66-5) exposure. VPA was dissolved in E3 medium and prepared as a 10 mM stock solution and diluted when used. In brief, after collection of the zebrafish embryos, they were immediately transferred into 6-well cell culture plates, about 30 embryos and 6 ml VPA per plate, kept in a 28.5°C incubator from 2 h postfertilization to 4.5 dpf, then washed with E3 twice, and transferred to a fry incubator (Hai Sheng) and fed with straw worms. The feeding was mixed with brine shrimp at 7 dpf, and the bottom was cleaned every day to maintain the water quality. The locomotion tests were performed at 14 dpf, and then the larval head sample was collected.

### Behavioral profiling

The behavioral assays were conducted by the DanioVision apparatus (Noldus, The Netherlands). When zebrafish are fed to 14 dpf, transfer them individually into 24-well plates. Behavioral testing was performed between 11:00 and 16:00 through the DanioVision system, which maintains a constant circulated water bath temperature at 29°C. High activity is defined as more than 80% change in the pixels of the targeted larva during one frame, which is defined and measured by the built-in algorithm. All the behaviors were tracked at 60 frames per second.

The setting of the locomotion test is 10 min of habituation for the dark environment, followed by 10 min of data collection in the dark condition. The dark-light transition locomotion experiment was conducted, followed by 2 min of dark and 2 min of light alternate testing. The light test was conducted like the dark test, with a 10-min adaptation and a 10-min light test. All kinds of information are recorded, like distance, velocity, meander, angular velocity, activity, and circulation times.

### Head tissue preparation

The 14 dpf wild-type larvae were anesthetized in tricaine, then the head was severed from the jaw to the area where the hindbrain meets the spinal cord, and then the eyes were separated following the protocol by Shainer *et al.* (31). Tissue was collected and washed with PBS three times in the tube and placed on ice, then the PBS was removed, and the weight of each tube was precisely 50 mg, and it was immediately stored in the centrifuge at −80°C for temporary preservation.

### Quantitative real-time PCR analysis

About 10 zebrafish brains at 14 dpf were collected in a tube. The total RNA extraction was conducted by the Trizol method, and the complementary DNA was synthesized from purified RNA using Takara PrimeScript™ RT reagent kit (Perfect Real Time). The quantitative PCR (qPCR) was performed using the SGExcel Ultra SYBR Mixture (Shenggong; B532957) in LightCycler® 480 Instrument II (Roche) with the following conditions: activation at 50°C for 2 min, denaturation at 95°C for 3 min, followed by 40 cycles of amplification consisting of a 15 s at 95°C, 1 min at 60°C with 1.6°C/min per step, 15 s at 95°C with 0.15°C/min, imaging the plate after every extension at 72°C for 1 min.

Relative expression values were determined using the comparative Ct (ΔΔCt) method, and transcript abundances were normalized to beta-actin transcript abundance. Primer sequences are given in supplemental Table S1.

### Metabolite extraction

Approximately 400 larvae (50 for each replicate, n = 8) were collected for metabolite extraction. After thawing and grinding the samples, hydrophilic compounds were extracted using 70% methanol-water containing an internal standard. The extracts were vortexed and centrifuged, and the supernatant was collected for instrument analysis. The hydrophobic material was added to the lipid extraction solvent containing the internal standard mixture and vortexed, then water was added followed by vortexing and centrifugation. The supernatant was aspirated, concentrated and added to the lipid complex solution and vortexed and centrifuged, and the supernatant was collected for instrument analysis.

### UPLC-QTRAP-MS/MS-based metabolomics analysis

Hydrophilic sample extracts were analyzed using an LC-ESI-MS/MS system (ultra-performance liquid chromatography [UPLC], ExionLC AD; MS; QTRAP® System). The analytical conditions were as follows: UPLC: column, Waters ACQUITY UPLC HSS T3 C18 (1.8 μm, 2.1 mm × 100 mm) at 40°C. The flow rate was 0.4 ml/min, and the injection volume was 2 μl. Mobile phase A was water (0.1% formic acid), and phase B was acetonitrile (0.1% formic acid). The gradient program was 95:5 v/v at 0 min, 10:90 v/v at 11.0 min, 10:90 v/v at 12.0 min, 95:5 v/v at 12.1 min, and 95:5 v/v at 14.0 min.

Linear ion trap (LIT) and triple quadrupole (QQQ) scans were conducted using QQQ-LIT mass spectrometer (QTRAP). The QTRAP® LC-MS/MS system includes an ESI Turbo Ion-Spray interface operating in positive and negative ion modes, controlled by Analyst 1.6.3 software (Sciex). The ESI source operation parameters were as follows: source temperature 500°C; ion spray voltage 5,500 V (positive) and −4,500 V (negative); ion source gas I, gas II, and curtain gas were set at 55, 60, and 25.0 psi, respectively; the collision gas (collision-associated dissociation) was high. Instrument tuning and mass calibration utilized 10 and 100 μmol/l polypropylene glycol solutions in QQQ and LIT modes, respectively. Multiple reaction monitoring (MRM) transitions were monitored for each period based on metabolite elution.

Hydrophobic sample extracts were analyzed using an LC-ESI-MS/MS system (UPLC, ExionLC AD; MS; QTRAP® System). The analytical conditions were as follows: UPLC: column, Thermo Accucore™ C30 (2.6 μm, 2.1 mm × 100 mm i.d.). Mobile phase A was acetonitrile/water (60/40, v/v, 0.1% formic acid, 10 mmol/l ammonium formate), and phase B was acetonitrile/isopropanol (10/90 v/v, 0.1% formic acid, 10 mmol/l ammonium formate). The gradient program is A/B (80:20, v/v) at 0 min, 70:30 v/v at 2.0 min, 40:60 v/v at 4 min, 15:85 v/v at 9 min, 10:90 v/v at 14 min, 5:95 v/v at 15.5 min, 5:95 v/v at 17.3 min, 80:20 v/v at 17.3 min, and 80:20 v/v at 20 min. The flow rate was 0.35 ml/min at 45°C, and the injection volume was 2 μl. The effluent was connected to an ESI-QQQ-LIT (QTRAP)-MS.

LIT and QQQ scans were obtained using QTRAP® LC-MS/MS System with an ESI Turbo Ion-Spray interface, operating in positive and negative ion modes and controlled by Analyst 1.6.3 software. The ESI source operation parameters were as follows: ion source, turbo spray; source temperature 500°C; ion spray voltage 5,500 V (positive), −4,500 V (negative); ion source gas 1, gas 2, and curtain gas were set at 45, 55, and 35 psi, respectively; the collision gas (collision-associated dissociation) was medium. Instrument tuning and mass calibration were carried out using 10 and 100 μmol/l polypropylene glycol solutions in QQQ and LIT modes, respectively. QQQ scans were acquired as MRM experiments with collision gas (nitrogen) set to 5 psi. Declustering potential and collision energy for individual MRM transitions were done with further declustering potential and collision energy optimization.

### Nile red staining and confocal imaging

Zebrafish brain cryosections were followed by the procedures (32). The zebrafish brain was fixed with 4% paraformaldehyde at room temperature for 2 h, washed three times with PBS, and then dehydrated overnight using a gradient of 10–30% sucrose solution until the fish sank to the bottom. Soak the brains with OCT (Sakura, Tissue-Tek OCT Compound, #4583), then embed them, and store at −80°C for 1 h for cryosectioning. The thickness should be controlled at 15–20 μm. Rewarm the frozen sections for 3 min, then fix at room temperature for 20 min. Wash three times with 1× PBS, then incubate in staining solution for 10 min in the dark. Wash with 1× PBS twice and cover the section with S2100 mounting medium.

Zebrafish whole brain staining was followed by the procedures (33, 34). The brain was washed three times with PBS and then fixed with 4% paraformaldehyde at 4°C for 12 h. Washed three times with PBS, then supplemented with Nile red to the staining solution, and then placed in the dark for 30 min. Washed three times with PBS and embedded in the low-melt agarose for imaging.

Confocal imaging was conducted using the Zeiss confocal microscope (LSM880), with the ZEN2012 software (Zeiss).

### Statistical analysis

The raw data were processed by the Analyst 1.6.3 software. The metabolites of the samples were analyzed qualitatively and quantitatively by MS based on local metabolic databases. The MultiQuant software (SCIEX, version 3.0) was used for peak integration and calibration.

Unsupervised principal component analysis was performed by the statistics function prcomp within R (www.r-project.org). The data were unit variance scaled before unsupervised principal component analysis. The results of the hierarchical cluster analysis (HCA) for both samples and metabolites were displayed as heatmaps with dendrograms. The Pearson's correlation coefficients between samples were determined by using the cor function in R and presented as heatmaps. Both HCA and Pearson's correlation coefficient were performed using the R package ComplexHeatmap. For HCA, the normalized signal intensities of metabolites were visualized as a color spectrum, with unit variance scaling.

The fluorescence intensity of confocal imaging was calculated with ImageJ (version 1.8.0). The confocal imaging was converted to 8-bit grayscale and processed with z-stack projection using either maximum intensity projection or SUM intensity projection. Subsequently, the auto threshold is used to count the fluorescence intensity of the target area, and finally, to measure the area selected, the mean intensity values or intensity values are used for statistics. Data were analyzed with Student’s *t*-test for comparing the two independent groups. The one-way ANOVA was used to compare three or more independent groups. A *P* value of less than 0.05 was considered statistically significant.

## Results

### Establishment and validation of a zebrafish model for autism

**Fig. 1:**
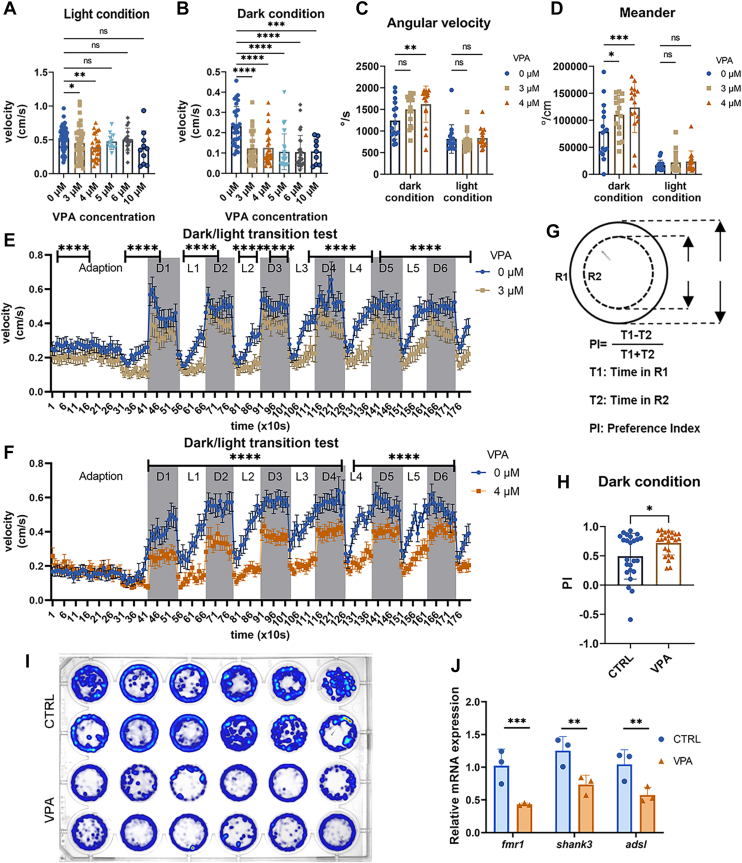
Zebrafish exposed to VPA exhibit a significant reduction in locomotor activity and display anxious behavior. A and B: The velocity (cm/s) analysis in the light and dark conditions was conducted on 14 dpf zebrafish exposed to VPA treatment, with concentrations ranging from 0 μΜ to 10 μM. n = 48 for 0 μΜ to 6 μΜ, n = 24 for 10 μΜ. C and D: The angular velocity (°/s) and meander (°/cm) in both dark and light conditions were observed with 3 μM and 4 μΜ VPA. n = 30. E and F: Velocity (cm/s) analysis during the transition test between dark and light conditions was conducted with 3 μM and 4 μΜ VPA. The shaded regions represent dark periods, whereas the unshaded regions indicate light periods. The transition of dark and light phases was repeated six times. n = 48 for each group. G: The schema of the division of R1 and R2 in one hole (top) and the formula to calculate the preference index (PI) value (bottom). H: The PI in the dark condition. n = 24, Student’s *t*-test. I: Heatmap of locomotion in the dark condition in a 24-well plate, the upper lines A and B are CTRL groups, and the C and D lines represent the VPA-treated groups. J: The qPCR result of neuronal development genes and ASD risk genes. adsl, adenylosuccinate lyase; fmr1, fragile X mental retardation; shank3, multiple ankyrin repeat domain protein. n = 3, Student’s *t*-test. Data are shown as mean ± SEM. ns, not significant. ∗*P* < 0.05, ∗∗*P* < 0.01, ∗∗∗*P* < 0.001, and ∗∗∗∗*P* < 0.0001.

**Supplementary Figure 1:**
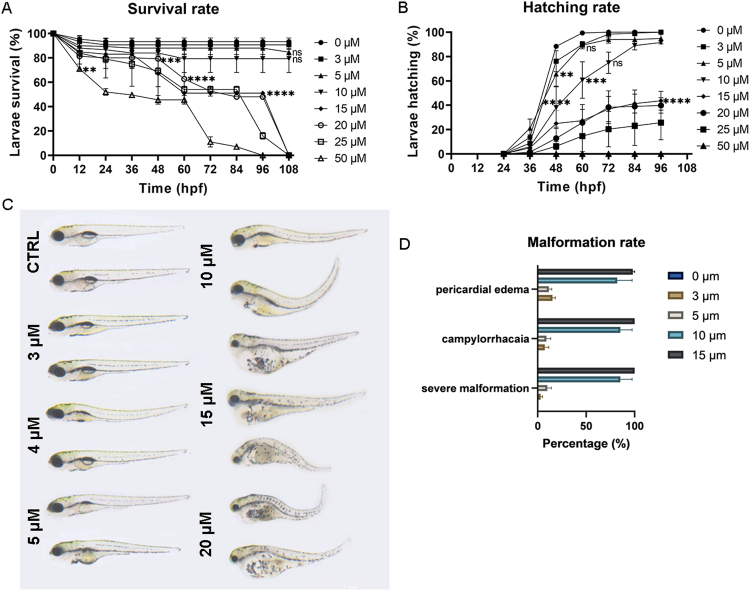
**High concentrations of VPA exposure cause an aberrant phenotype in zebrafish.** (A) The survival rate of different concentrations of VPA exposure, ranging from 0 μM to 50 μM from 0 hours post fertilization (hpf) to 108 hpf. (B) The hatching rate from 0 μM to 50 μM of VPA exposure. (C) The effect of VPA on zebrafish morphological structure. Lateral view. Scale bar, 2 mm. (D) The malformation rate included the pericardial edema, campylorrhacaia and overall assessment in VPA exposure from 0 μM to 15 μM. Data are collected from three independent treated groups and analyzed using one-way ANOVA. Each n=50. ns, not significant. ∗ *p* < 0.05, ∗∗ *p* < 0.01, ∗∗∗ *p* < 0.001, and ∗∗∗∗ *p* < 0.0001.

Zebrafish were subjected to varying concentrations of VPA, ranging from 3 μM to 50 μM (35, 36, 37), during the critical period of neural tube development and closure, specifically from the cleavage stage (0.75–2.25 h) to 4.5 dpf. The groups exposed to 3 μM and 4 μM concentrations exhibited only minor delays in incubation and development (Supplemental Fig. S1). To investigate the potential impact of VPA on behavioral profiling in zebrafish (38, 39), treatment groups with concentrations of 3 μM, 4 μM, 5 μM, and 6 μM were selected for the assessment of motor behavior. In the velocity test of both light and dark conditions, zebrafish exposed to 4 μM demonstrated a significant reduction in velocity within the light condition, whereas all tested concentrations resulted in a notable decrease in velocity within the dark field (Fig. 1A, B). In addition, there was a significant decline in angular velocity (quantified as the change in movement direction per unit time [°/s]), and meandering behavior was observed between the 3 μM and 4 μM exposure groups (Fig. 1C, D), which related to autism-like behaviors (40, 41). In the dark-light shift locomotion test, zebrafish in the 4 μM group exhibited diminished locomotor activity during the transition from light to dark (Fig. 1E, F). These findings indicate 4 μM as the optimal concentration for subsequent experimental investigations and validate the zebrafish model for ASD.

The preference index (time in outer ring - time in the center ring) for movement time in the outer zone was evaluated (Fig. 1G). The VPA treatment group allocated more time in the outer ring (R1) compared with the control (CTRL) group, indicating a significant alteration in the preference index (*P* = 0.016) (Fig. 1H). In addition, trajectory heatmaps further confirmed that the anxiety levels of zebrafish in the VPA treatment group were elevated (Fig. 1I), and the VPA treatment group spent longer times in the outer circle compared with the CTRL group.

To further assess the validity of this model, several ASD-associated genes were examined, including fragile X mental retardation 1 (*fmr1*), a key regulatory gene in the CNS (42), multiple ankyrin repeat domain protein (*shank3*), which encodes a scaffolding protein that is involved in the functionality of α-amino-3-hydroxy-5-methyl-4-isoxazolepropionic acid receptor, spine stabilization, and synaptic plasticity within excitatory glutamatergic synapses (43), and adenylosuccinate lyase (*adsl*), a gene linked to neurometabolic disorders (44). These genes have been previously identified and classified as high-risk ASD genes in the SFARI GENE database, and they are also associated with the advancement of neuronal development. Notably, their expression levels were significantly reduced in the group exposed to 4 μM (Fig. 1J).

### Metabolite profiles and metabolic pathways between VPA and CTRL groups

**Fig. 2:**
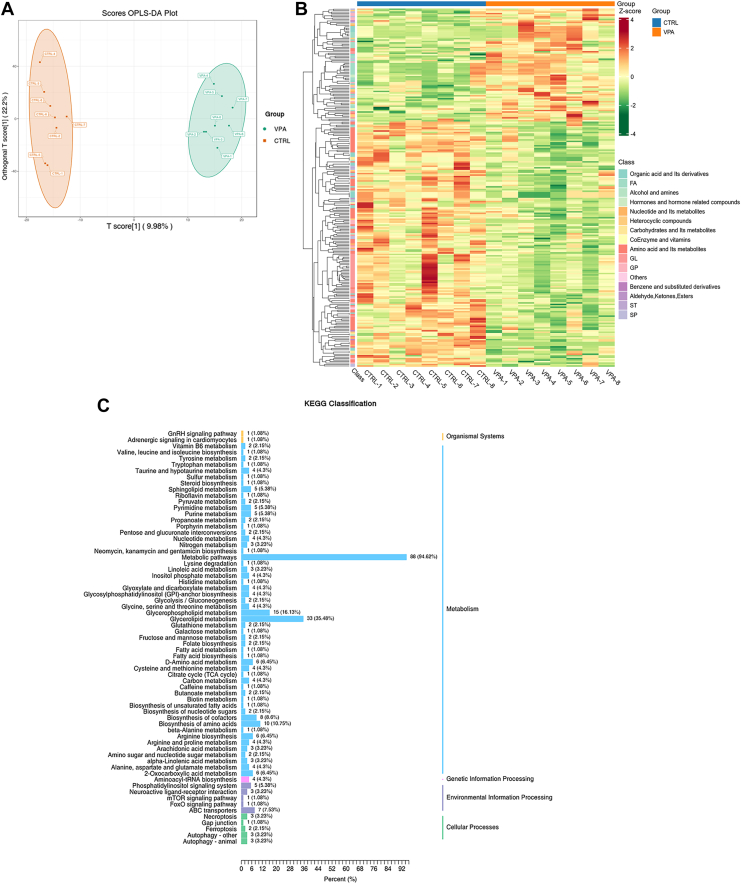
The overall difference between VPA and CTRL groups and the Kyoto Encyclopedia of Genes and Genomes classification of DMs. A: The OPLS-DA plot shows the differences between the two groups of zebrafish brain metabolisms at 14 dpf. The *x*-axis indicates the predicted principal component, and the direction along the *x*-axis shows the gap between groups. The *y*-axis indicates the orthogonal principal component, and the direction along the *y*-axis shows the gap within groups. n = 8. B: The *x*-axis of heatmap is the name of the sample, and *y*-axis is the DM information, and the colors were filled with different values obtained from the standardized treatment of different relative contents (red means high content, and green means low content). This heatmap only analyzes the metabolite cluster; the cluster line on the left side of the figure is the metabolite cluster line. C: The Kyoto Encyclopedia of Genes and Genomes metabolic pathway shows the number and ratio of DMs annotated to each pathway. OPLS-DA, orthogonal partial least squares discriminant analysis; SP, sphingolipid; ST, sterol lipid.

**Supplementary Figure 2:**
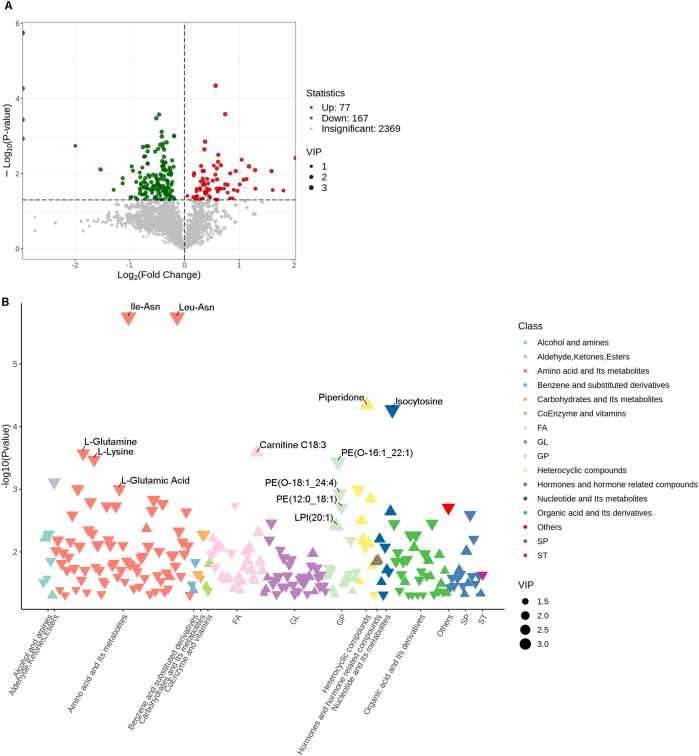
**Intergroup differences in Metabolite profiles between VPA and CTRL groups revealed by principal component analysis.** (A) The volcano plot displays the differential metabolites in VPA filtered by univariate analysis. Each point represents a metabolite: green points indicate downregulated differential metabolites, red points indicate upregulated differential metabolites, and gray points indicate metabolites detected but with insignificant differences. The x-axis represents the logarithm of the multiplicity of the differences in the relative contents of a metabolite between the two groups of samples (log_2_FC). The larger the absolute value of the horizontal coordinate, the larger the difference in the relative content of the substance between the two groups of samples. (B) The Differential Substance Classification Scatterplot combines the results of the differential situation of substances and the category to which the substances belong, from which it is possible to visualize which types of substances are detected the most, and the difference of each type of substance in the comparison group. The horizontal coordinates of the graph below are the metabolite categories, one color represents a class of substances, and the vertical coordinates are the significance of the differences, with a -log_10_ treatment for the P value. Positive triangles represent up-regulated substances, inverted triangles represent down-regulated substances, and the size of the triangles represents the size of the VIP values. FA, fatty acid; GL, glycerolipids; GP, glycerophospholipids; ST, sterol lipids; SP, sphingolipids.

**Supplementary Figure 3:**
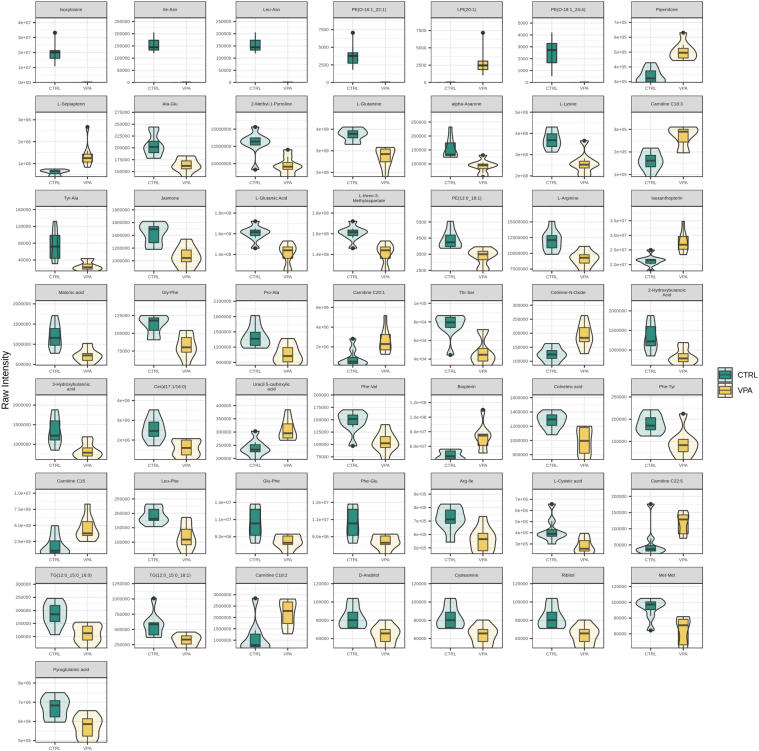
**Violin plot of the top 50 metabolites with the highest VIP values.** The horizontal coordinate is the grouping and the vertical coordinate is the relative content of the differential metabolite (raw peak area). The box shape in the center indicating the interquartile range, the thin black line extending from it representing the 95% confidence interval, and the black horizontal line right in the middle being the median, and the shape of the outer part indicating the density of the distribution of the data. PE, phosphatidylethanolamine; LPI, lysophosphatidylinositol; Cer, ceramides.

We employed a UPLC-MS/MS-based comprehensive metabolomic methodology to examine the effects of VPA on the neurodevelopment of zebrafish and to identify various differential metabolites (DMs). The orthogonal partial least squares discriminant analysis revealed a clear separation between the metabolic profiles of brain tissue of the VPA and CTRL groups (Fig. 2A). A comparative analysis of the DMs, based on fold changes, was performed between the CTRL and VPA groups, with the findings illustrated in a volcano plot (Supplemental Fig. S2A). This analysis identified 167 significant metabolites exhibiting a downward trend and 77 metabolites exhibiting an upward trend. Hierarchical clustering of these DMs further highlighted distinct patterns between groups (Fig. 2B). The classification of the top DMs was shown in the Differential Substance Classification Scatterplot (Supplemental Fig. S2B); there were 75 DMs belonging to amino acids and their metabolites, 35 to glycerolipids (GLs), 30 to organic acids and their derivatives, 29 to FAs, 18 to sphingolipids, 17 to glycerophospholipids (GPs), 10 to heterocyclic compounds, and the remainder included nucleotides, alcohols, amines, carbohydrates, benzene derivatives, coenzymes, vitamins, hormones, aldehydes, ketones, esters, sterol lipids, and other compounds. The top 50 DMs based on the Variable Importance in Projection (VIP) score are shown in the violin plot (Supplemental Fig. S3).

The application of Kyoto Encyclopedia of Genes and Genomes classification resulted in the identification of over 50 distinct metabolic pathways. GL metabolism (33 DMs, 35.48%), GP metabolism (15 DMs, 16.13%), and amino acid biosynthesis (10 DMs, 10.75%) were identified as the three most prominent pathways (Fig. 2C). The GL metabolism played a crucial role in the synthesis and breakdown of neutral lipids, comprising 28 TGs, 5 diglycerides, and 1 phosphatidic acid (PA). The GP metabolism was intricately linked to GL metabolism, including components such as diglyceride, phosphatidylethanolamine (PE), PA, phosphatidylcholine (PC), phosphatidylserine, and lyso-PC.

### Analysis of metabolites and gene expression of lipid metabolism pathway

**Fig. 3:**
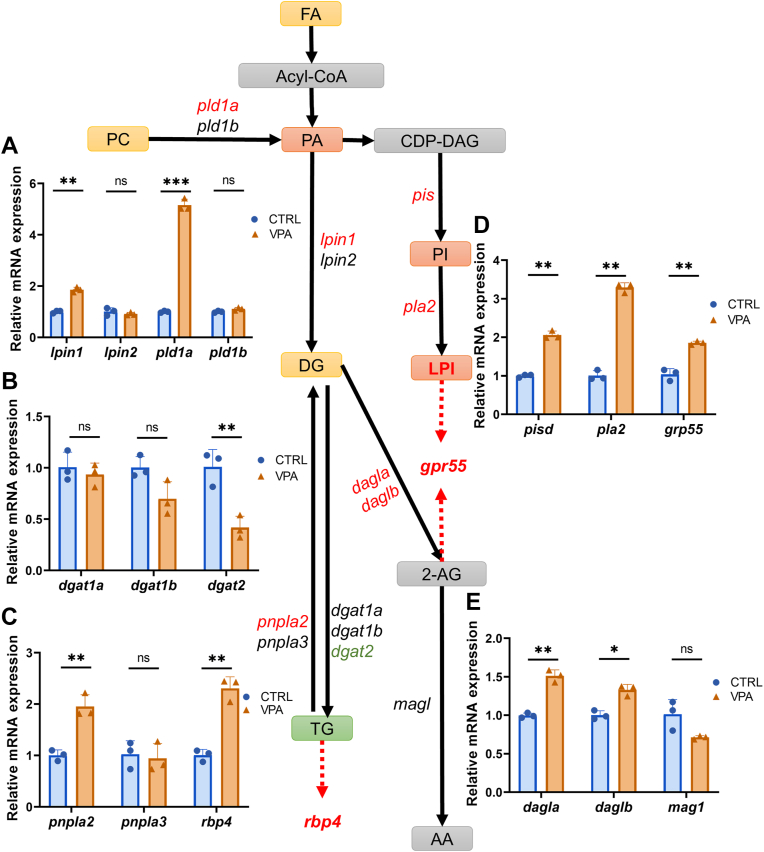
Lipid profiling shows a significant difference between the CTRL and VPA groups. Common changes in VPA-exposed groups are highlighted in green (for reductions), red (for increments), orange (for both reductions and increments), and gray (for not significant change or not detected). A–E: qRT-PCR analyzes the expression of the related genes of the metabolism in 14 dpf zebrafish head tissues. The expression genes in CTRL were normalized as 1 to calculate the relative levels of other groups, and β-actin was used as the internal standard. The red highlighted genes are upregulated, whereas the black genes show nonsignificant changes. AA, arachidonic acid; CDP-DAG, cytidine diphosphate DAG; DG, diglyceride; PI, phosphatidylinositol. n = 3, Student’s *t*-test. ns, not significant. ∗*P* < 0.05, ∗∗*P* < 0.01, and ∗∗∗*P* < 0.001.

The lipid metabolic pathway affected by VPA exposure is summarized in Figure 3. Based on the VIP score, there was a significant increase in certain metabolites within the group exposed to VPA. These metabolites were ranked in descending order of significance as follows: SM (d18:1/26:1), diacylglycerol (DAG) (18:0_18:1), phosphatidylserine (18:0_20:5), DAG (20:0_22:6), and FFA (20:0). Conversely, the metabolites that exhibited a decrease in rank included PE (12:0_18:1), PC (16:0_14:0), and lyso-PC (14:1/0:0). The majority of lipid metabolism is derived from FAs, which are ultimately transformed into SM, DAG, and lysophosphatidylinositol (LysoPI or LPI).

**Supplementary Figure 4:**
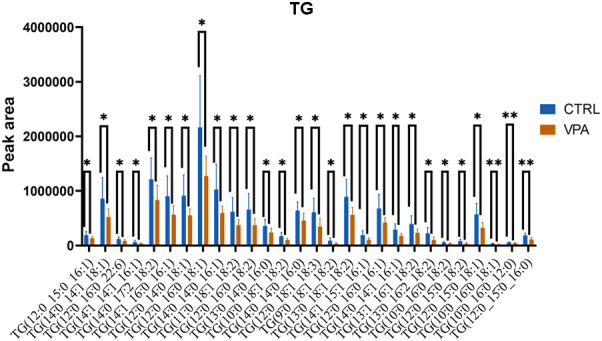
**The peak area of 28 TGs in metabolic results.** Data are analyzed using one-way ANOVA. ∗ *p* < 0.05 and ∗∗ *p* < 0.01.

The most significant modification observed in lipid metabolism was the widespread downregulation of TGs, with a total of 28 TGs identified as downregulated (Supplemental Fig. S4), of which three displayed substantial downregulation. The key enzyme genes involved in the TG synthesis pathway were examined using qPCR (Fig. 3A–C). The gene *dgat2* exhibited a decreasing trend, whereas *pnpla2* and the downstream gene associated with ASD, *rbp4*, were significantly upregulated. These changes in gene expression may primarily account for the notable downregulation of TGs. Retinol-binding protein 4 (RBP4) is hypothesized to function as an inflammatory neurotrophic adipokine capable of crossing the blood-brain barrier, which is frequently compromised in many individuals with autism (45, 46).

**Fig. 4:**
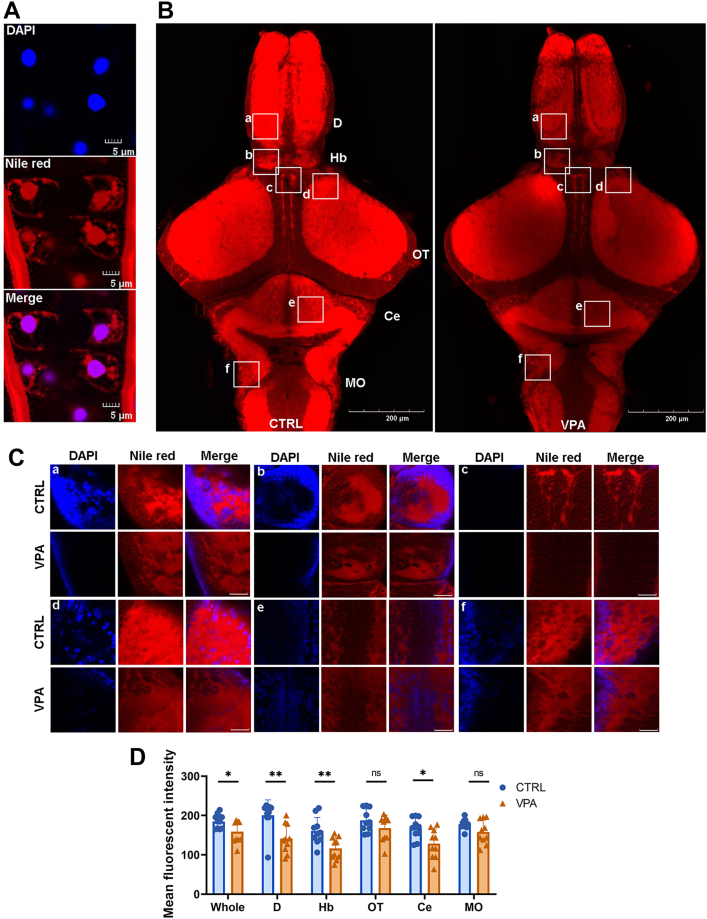
Nile red staining indicates the distribution of TG in the brain and the change in VPA groups. A: The distribution of TG at the cellular level imaging of CTRL was conducted with the confocal microscope. DAPI stains the cell nucleus, Nile red represents TG, and the merge represents DAPI with Nile red fluorescence. The scale bar represents 5 μm. B: The fluorescence reduces in the VPA treatment group, especially in the selected places. The confocal image was conducted through Z-stack, maximum intensity projection. The scale bar represents 200 μm. C: Local zoom in the selected places in (B), a, V; b, Hb; c, OT; e, Ce; and f, MO. The CTRL group is at the top, and the treatment group is at the bottom. The scale bar represents 10 μm. D: Mean fluorescence intensity counts for the whole brain as well as the selected sections. n = 10, Student’s *t*-test. ns, not significant. ∗*P* < 0.05 and ∗∗*P* < 0.01.

LPI is a metabolite derived from PA, a bioactive lipid that has been recognized for its direct neuroprotective effects (47). The expression levels of key enzyme genes involved in the LPI metabolic pathway, specifically *pis*, *pla*, and G-protein-coupled receptor 55 (*gpr55*), were found to be significantly upregulated (48) (Fig. 3D). Research indicates that GPR55 plays a role in mediating antianxiety effects, and its activation may enhance the neuroprotective outcomes associated with hippocampal neurogenesis by activating Gαq and its downstream signaling pathways. In addition, the expression of *dagla/b*, an enzyme critical for the regulation of the 2-arachidonoylglycerol (2-AG) signaling pathway within the CNS, was also observed to be upregulated. Conversely, the expression of the enzyme gene *magl*, which is responsible for the catabolism of 2-AG, remained unchanged (Fig. 3E). The observed downregulation of TGs suggests that a greater proportion of DAG may be converted into 2-AG, a process also associated with *gpr55*. To examine the distribution of TGs within the zebrafish brain, we employed the Nile red staining method. Our results indicated that TGs were primarily concentrated in the nucleus, nuclear membrane, and cell membrane (Fig. 4A). Following treatment with VPA, we observed a notable decrease in overall fluorescence across several brain regions, including the pallium (dorsal telencephalon, D), subpallium (ventral telencephalon, V), habenula (Hb), optic tectum (OT), corpus cerebelli (Ce), and medulla oblongata (MO) (Fig. 4B, C), with the D (*P* = 0.0069) and Hb (*P* = 0.0015) exhibiting the most pronounced difference (Fig. 4D).

### Analysis of metabolites and gene expression in glutamate signaling pathway

**Fig. 5:**
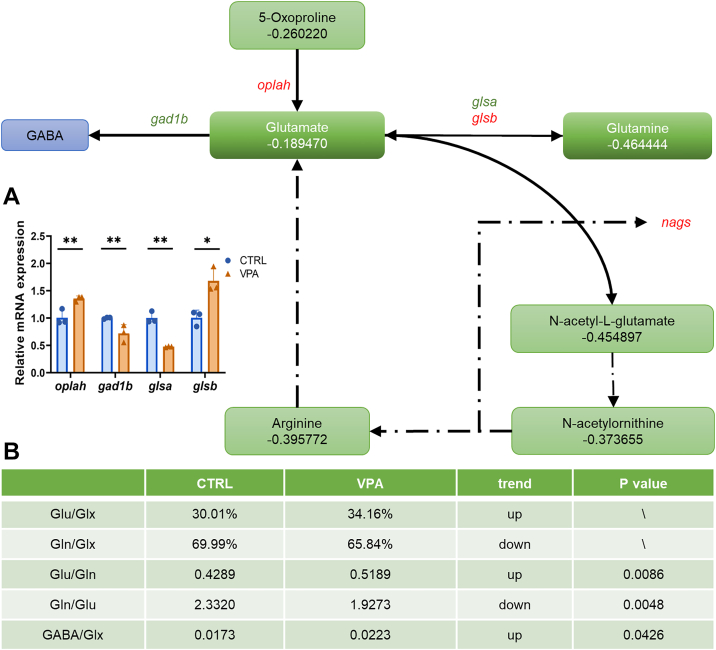
Main disturbed metabolic pathways of the relevant nonlipid metabolites between VPA and CTRL zebrafish. Common changes in VPA-exposed groups are highlighted in green (for reductions), red (for increments), gray (for not significant change), and blue (for not detected). The enzyme genes are labeled on the arrow. The solid arrow means direct reaction, and the long dotted arrow mark means the indirect reaction. The number under metabolisms is the VPA versus CTRL Log_2_FC value. A: qRT-PCR analyzes the expression of the resource’s genes of the metabolism in 14 dpf zebrafish head tissues. The expression genes in CTRL were normalized as 1 to calculate the relative levels of other groups, and the β-actin was used as the internal standard. The red highlighted genes were upregulated, whereas the green highlighted genes were downregulated, and the black highlighted genes were with nonsignificant changes. B: The Glu-Glx, Gln-Glx, Glu-Gln, Gln-Glu, and GABA-Glx (Glu + Gln) ratios were calculated in the table. ∗*P* < 0.05 and ∗∗*P* < 0.01.

The glutamate signaling pathway encompasses the metabolic process involving glutamate and glutamine (Gln). Dysregulation of glutamate has been linked to impairments in energy metabolism and neuronal function within the nervous systems of individuals diagnosed with ASD (49, 50). MS analyses indicated that the levels of glutamate, Gln, its precursor 5-oxy-proline, and the downstream metabolite *N*-acetyl-l-glutamate (NAG) in the brains of VPA-exposed zebrafish were significantly lower compared with the CTRL group (Fig. 5A). NAG is synthesized from glutamate and coenzyme A through the enzyme activity of NAG synthetase, which facilitates the production of NAG. This enzymatic reaction is predominantly supported by arginine, which was also found to be significantly diminished in the VPA-exposed group (Fig. 5A).

The primary mechanism for ammonia waste processing in the brain is the conversion of glutamate to Gln. This process is critical for preventing ammonia toxicity and mitigating synaptic glutamate accumulation, thereby reducing excitotoxicity. This interconversion of Glu and Gln is mediated by the enzymes glutaminase a (*glsa*) and glutaminase b (*glsb*). Furthermore, GABA, a key inhibitory neurotransmitter, is synthesized from glutamate via the action of glutamate decarboxylase (*gad1b*). qPCR analyses revealed a significant decrease in the expression of the *glsa* and *gad2b* genes, whereas a modest increase in *glsb* gene expression was noted. Numerous studies have established a strong correlation between neuropsychiatric disorders and an imbalance in the ratios of excitatory and inhibitory (E/I) neurotransmitters. Specifically, the concentrations of GABA and Glx (the sum of Glu and Gln) levels have been proposed as potential biomarkers for ASD and schizophrenia (51). In addition, an elevated plasma Glu-Gln ratio has been observed in children diagnosed with autism (52). MS findings indicated a significant increase in the Glu-Gln ratio in the group exposed to VPA (*P* = 0.0086), whereas the GABA-Glx ratio was recorded at 0.0173 in the CTRL group and 0.0223 in the VPA-exposed group (*P* = 0.0426), demonstrating a significant increase as well (Fig. 5B).

### Expression analysis of glutamic and GABAergic neurons in the brain

**Fig. 6:**
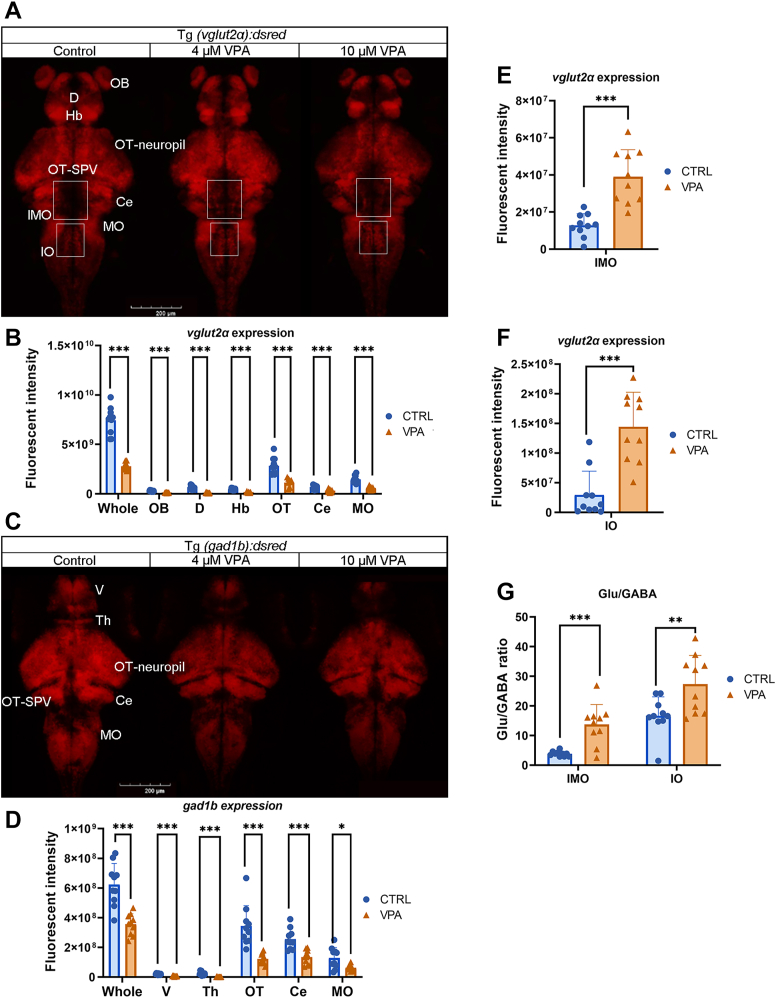
The distribution of glutamic and GABAergic neurons in the whole brain. A: The confocal living imaging of glutamic neurons in Tg(*vglut2α:DsRed*) at 14 dpf. The confocal image was conducted through Z-stack, SUM intensity projection. The scale bar represents 200 μm. B: Fluorescence intensity counts for the whole brain, OB, D, Hb, OT, Ce, and MO. n = 10. C: The confocal living imaging of GABAergic neurons in Tg(*gad1b:DsRed*) at 14 dpf. The confocal image was conducted through Z-stack, SUM intensity projection. The scale bar represents 200 μm. D: Fluorescence intensity counts for the whole brain, V, Th, OT, Ce, and MO. n = 10. E and F: The fluorescence intensity counts in IMO and IO. n = 10. G: The ratio of Glu and GABA in IMO and IO. n = 10. OB, olfactory bulb. The scale bar represents 200 μm. n = 10, Student’s *t*-test. ∗*P* < 0.05, ∗∗*P* < 0.01, and ∗∗∗*P* < 0.001.

In order to more precisely analyze the spatial expression relationship between glutamatergic and GABAergic neurons, transgenic zebrafish strains, Tg*(vglut2α:DsRed)* and Tg*(gad1b:DsRed)*, which express fluorescent markers for glutamate and GABA, respectively, were subjected to VPA treatment at concentrations of 4 μM and 10 μM. Subsequent whole-brain imaging was conducted at 7 dpf. The results indicated a pronounced gradient in fluorescence intensity among glutamatergic and GABAergic neurons across the CTRL group and the two VPA-exposed groups (Fig. 6A, C). Notably, in the VPA-exposed groups, there was a significant reduction in expression levels within the forebrain and midbrain regions, including the olfactory bulb (OT), central nucleus (Ce), and MO. In addition, a decrease in GABAergic neuron populations was observed in the posterior brain, whereas glutamatergic activity was markedly increased in specific localized areas (Fig. 6A–D).

Dysplasia of the OT (53) and the abnormal expression of glutamatergic neurons in the dorsal region (54, 55), as well as in the middle area of the stratum periventriculare (SPV), are intricately linked to the etiology of autism. The Hb, situated in the upper thalamus (Th), plays a crucial role in the processing of social information and the regulation of social behavior. Furthermore, it is associated with various human behaviors, including sleep and learning (56). In the VPA-exposed group, a notable decrease in the fluorescence intensity of glutamatergic neurons was observed throughout the entire Hb region. Conversely, there was a significant increase in the central region, whereas a marked decrease was noted in the olfactory bulb, D, and SPV regions (Fig. 6A, B).

The Th, located in the central brain region, is integral to various functions, including motor control, consciousness maintenance, sleep regulation, and other essential processes. It is also significantly associated with sensory disorders, involuntary movements, and disturbances in consciousness (57). The ventral telencephalon (V) is vital for embryonic and early developmental stages, as it is responsible for the generation of inhibitory neurons and interneurons, whereas region D is characterized by abundant GABAergic neurons (58). Following treatment with VPA, a pronounced reduction in fluorescence intensity was recorded in the Th and V of the *gad1b* transgenic zebrafish strain, rendering them nearly imperceptible (Fig. 6C, D).

The OT is a complex structure located in the midbrain that plays a crucial role in the processing and integration of various sensory inputs as well as in the regulation of behaviors, such as phototaxis, predation, and evasion from predators (53). In the VPA-exposed group, there was a notable reduction in both glutamatergic and GABAergic neurons at the OT site, particularly in the subgroup exposed to 10 μM, which exhibited a markedly irregular pattern and appeared structurally and dimensionally abnormal (Fig. 6A–D).

Previous studies have indicated that individuals with autism frequently display an atypical enlargement of the inferior olive (IO) and alterations in the distribution of glutamatergic neurons (59, 60). Following VPA administration, there was a significant increase in fluorescence intensity within the intermediate MO (IMO) and IO in the brains of *vglut2α* transgenic zebrafish, accompanied by a substantial alteration in the ratio of glutamate to GABA (Fig. 6E–G).

## Discussion

Metabolites serve as a fundamental component of an organism's phenotype, offering an enhanced understanding of biological processes and mechanisms that extend beyond the realms of genomics and proteomics. In this study, a VPA-induced zebrafish model of autism at 14 dpf was utilized to perform a full-spectrum metabolomic analysis. The model was validated through behavioral locomotor assays and quantification of ASD-associated risk genes (*fmr1*, *shank3*, and *adsl*), confirming its relevance to ASD. The metabolomic analysis indicated that in the VPA-exposed group, several essential amino acids, including 5-oxyproline, lysine, glutathione, l-glutamic acid, Gln, and l-lactic acid, were found to be downregulated. These observations are consistent with prior studies identifying similar metabolites in the serum or brain of individuals with ASD or animal models (61, 62), thereby reinforcing the validity of the VPA-induced zebrafish model.

The Kyoto Encyclopedia of Genes and Genomes attributes the DMs to the GL and GP metabolism as well as to the amino acid pathway. The GL metabolism related to the composition of cell membranes and membrane-associated signaling may undergo systematic remodeling (63). The GP metabolism is intricately linked to GL metabolism. These elements are fundamental to the structure of cell membranes, particularly in neuronal cells and myelin sheaths, influencing membrane fluidity, the function of membrane proteins, and the formation and fusion of synaptic vesicles (64). Emerging data increasingly highlight the critical role of lipid remodeling and transfer mechanisms as central regulators of CNS lipid homeostasis (65), and its dysregulation is directly implicated in neurodevelopmental disorders (66).

Amino acid biosynthesis, including of l-Gln, l-lysine, l-glutamic acid, l-arginine, which has been linked with several pathological conditions, also play an important role in several neurological diseases (67). Amino acid metabolic imbalances are common in ASD patients, which can affect neurotransmitter synthesis, neuronal excitatory/inhibitory balance, and mitochondrial function. For example, abnormal metabolism of key amino acids such as glycine, serine, glutamate, and Gln can directly interfere with *N*-methyl-d-aspartate receptor function and neurotransmission, which is closely related to the pathophysiological mechanisms of ASD (68, 69).

These metabolic disturbances are directly linked to the pathogenesis of neurodevelopmental disorders. The following discussion will elaborate on these two major thematic directions.

### Relationship between brain lipids and *rbp4* and *gpr55* genes

Lipids play an essential role in brain development (70, 71), and disruptions in lipid homeostasis are increasingly recognized as a hallmark of ASD (4). Investigations into the relationship between autism and lipids, particularly PUFAs, suggest that infants lacking breastfeeding or PUFA supplementation face a significantly heightened risk of developing developmental autism, whereas EPA supplementation may mitigate this risk (72). Chen *et al.* (45) identified a significant increase in serum RBP4 levels and a marked decrease in TG, both of which were closely linked to the severity of autism. In addition, Martin *et al.* observed that neuronal activity associated with Rbp4-cre was heightened in mice harboring mutations in ASD risk genes *Chd8* or *Grin2b*, indicating that the activation of RBP4 may contribute to dysregulated migration and anxiety-like behaviors (73, 74). Moreover, TG levels were significantly diminished in the brains of autistic zebrafish generated through VPA treatment, representing a notable alteration in lipid metabolism, while the expression of the *rbp4* gene was significantly upregulated (Fig. 3C).

GPR55 mRNA is highly expressed in the mammalian brain and plays a role in various regulatory functions within this organ (48). Several studies have suggested that the activation of GPR55 in the hippocampus may be implicated in the modulation of anxiety-like behaviors (75, 76, 77). VPA has been shown to upregulate the extracellular signal-regulated kinase (ERK) signaling pathway. Notably, LPI can activate the CNS regulatory factor GPR55, which subsequently activates ERK1/2, potentially contributing to the pathophysiology of autism (78, 79, 80). In the VPA-induced zebrafish model, both LPI and *gpr55* gene expression were significantly upregulated (Fig. 3E). Thus, it is hypothesized that VPA induction may enhance the synthesis of LPI, increase the expression of *gpr55*, and subsequently activate the ERK1/2 signaling pathway, potentially contributing to the development of autism.

### The balance between Glx and GABA compared with the expression of TG

The E/I imbalance represents a prominent hypothesis regarding the pathogenesis of autism (81, 82, 83). The disruption of the glutamate-Gln cycle has been closely associated with various neurological disorders (84, 85). In VPA-induced zebrafish, there was a significant reduction in glutamate and Gln levels, accompanied by a notable increase in the ratios of Glu-Gln and GABA-Glx (Fig. 5A, B). These findings suggest a disturbance in their regulatory pathway governing glutamate metabolism. In vivo imaging was conducted to assess the activity of excitatory glutamatergic neurons and inhibitory GABAergic neurons in the brains of zebrafish subjected to 4 μM VPA. The results revealed a general decrease in the fluorescence activity of glutamatergic neurons when compared with the untreated group, which is consistent with the observed overall reduction in glutamate levels. This diminished fluorescence of glutamatergic neurons was primarily noted in the V, Ce, and OT regions, wheraes an increase in fluorescence was recorded in the central region of the MO (IMO) and the IO nucleus. Conversely, the fluorescence intensity of GABAergic neurons decreased across most regions, particularly in the V, D, OT, and MO areas. This observation indicates a significant E/I imbalance between glutamatergic and GABAergic neurons in the IMO and IO regions (Fig. 6). Furthermore, metabolomic analysis aimed at detecting GABA neurotransmitters in the brains of VPA-treated zebrafish did not reveal significant alterations; however, a marked reduction in fluorescence intensity of GABAergic neurons was observed in the OT and SPV regions of the brain in transgenic zebrafish. This suggests that the E/I imbalance in specific brain regions may contribute to a decrease in neuronal excitability, which is associated with behaviors characteristic of autism.

Lipids constitute approximately 60% of the brain and are essential for maintaining normal brain function and preventing neurological disorders. Environmental neurotoxicity disrupts cell homeostasis through lipid peroxidation, leading to nerve damage (86). VPA, as a short-chain FA derivative, may directly or indirectly affect the lipid metabolic environment (including TG) in zebrafish, which is similar to some plant extracts, and can create different activities and functions after modification (87). Nile red staining in the brains of zebrafish treated with VPA indicated a significant reduction in lipid content within the Hb and OT, paralleled by decreased fluorescence intensity of both excitatory glutamatergic and inhibitory GABAergic neurons in these regions (Figs. 4 and 6A, C). Comparative analysis of the distribution of glutamic acid and GABAergic neurons with TG staining results revealed that the fluorescence of all three was downregulated in the OT and MO regions of VPA-treated zebrafish. Previous studies have indicated that lipid activity in the brain influences the expression of the glutamate transporter and excitatory amino acid transporter 2 as well as the excitotoxic effects of glutamate (88). Based on the detection results, it can be inferred that the reduction of TG in the cortex, along with the decline in glutamatergic and GABAergic neurons, may disrupt signal transmission, leading to the interruption of synchronous feedback signals in the brain. This disruption could impair normal motor behaviors and feedback mechanisms in VPA-treated zebrafish, potentially contributing to the development of autism.

### The relationship between other lipids and ASD

Among the DMs, there are 17 DMs belonging to GLs, two PEs were downregulated, PE (12:0_18:1) (VIP = 2.36, *P =* 0.002) and PE (17:0_20:5) (VIP = 2.13, *P* = 0.02), whereas one PE was upregulated, PE (22:5_22:6) (VIP = 1.84, *P =* 0.03). Similarly, two PCs showed downregulation, PC (16:0_14:0) (VIP = 2.03, *P* = 0.007) and PC (14:0_14:0) (VIP = 2.01, *P* = 0.02). PC and PE represent the most abundant phospholipids in the cellular membranes, and the ratio of PC and PE can alter the physical properties of the membrane bilayer (89, 90), inducing “lipid bilayer stress,” which triggers/exacerbates ER stress and the unfolded protein response (91) and affects the stability of transmembrane proteins and calcium homeostasis. Research reports indicate that reductions in PC and PE have been observed in the prefrontal cortex and serum of children with ASD, accompanied by a decrease in the activity of mitochondrial electron transport chain complexes, especially in the Ce and prefrontal cortex (92, 93). These results suggest a plausible mechanistic connection between the induction of autism and dysregulation of phospholipid metabolism (94).

In summary, this study investigated the VPA-induced ASD zebrafish model utilizing full-spectrum metabolomic analysis and in vivo fluorescence imaging to assess E/I neuronal populations within the brain. The findings revealed a significant disruption in the E/I balance, particularly within the IMO and IO regions. In addition, the metabolic pathway associated with glutamate was found to be altered, and there was a noted decrease in the levels of TG in the brain. Moreover, the observed reduction of glutamatergic and GABAergic neurons in the pallium was identified as a primary contributor to the manifestation of autism. These results are expected to provide a foundation for further exploration of the metabolic process linked to ASD and enhance the diagnostic approaches for the disorder. Future work will focus on elucidating the mechanistic links between lipid metabolites, particularly TGs, and neurotransmitter systems, with the goal of identifying novel biomarkers for autism. Ultimately, this research could facilitate the translation of these metabolic findings into clinical applications, such as using magnetic resonance spectroscopy to detect region-specific metabolic alterations in the brains of ASD patients for diagnostic purposes.

## Data availability

The data that support the findings of this study are available from the corresponding author upon reasonable request.

## Supplemental data

This article contains supplemental data.

## Conflict of interest

The authors declare that they have no conflicts of interest with the contents of this article.
