# Supplementary material for: Dysregulation of Glu-GABA and reduction of triglycerides contribute to valproic acid-induced autism model in zebrafish
Source: J Lipid Res. 2025 Sep 23;66(11):100911. doi: 10.1016/j.jlr.2025.100911 (PMC12605571; doi:10.1016/j.jlr.2025.100911)
Supplement: Supplementary Material [file mmc2.docx]

**Title**

**Dysregulation of Glu/GABA and reduction of triglycerides contribute to valproic acid-induced autism model in zebrafish**

**Supplementary Information**

**
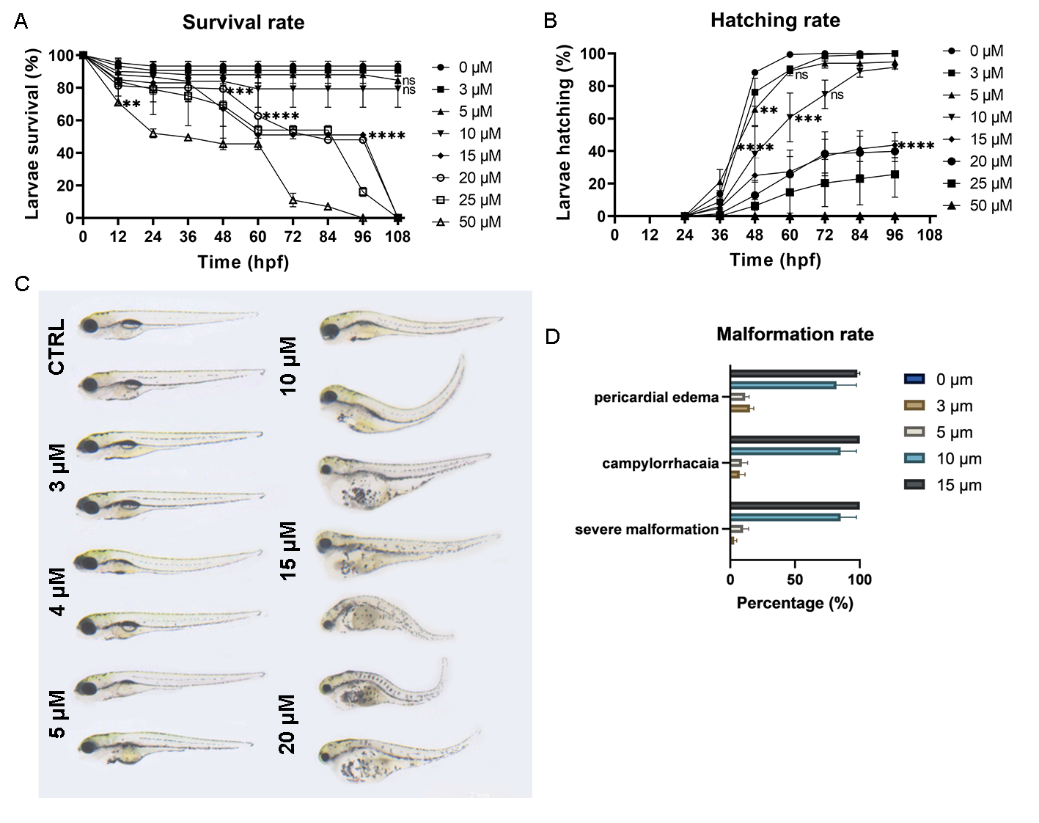
**

**Supplementary Figure 1. High concentrations of VPA exposure cause an aberrant phenotype in zebrafish.**

(A) The survival rate of different concentrations of VPA exposure, ranging from 0 μM to 50 μM from 0 hours post fertilization (hpf) to 108 hpf.

(B) The hatching rate from 0 μM to 50 μM of VPA exposure.

(C) The effect of VPA on zebrafish morphological structure. Lateral view. Scale bar, 2 mm.

(D) The malformation rate included the pericardial edema, campylorrhacaia and overall assessment in VPA exposure from 0 μM to 15 μM.

Data are collected from three independent treated groups and analyzed using one-way ANOVA. Each n=50. ns, not significant, * *p* < 0.05; ** *p* < 0.01; *** *p* < 0.001; **** *p* < 0.0001.


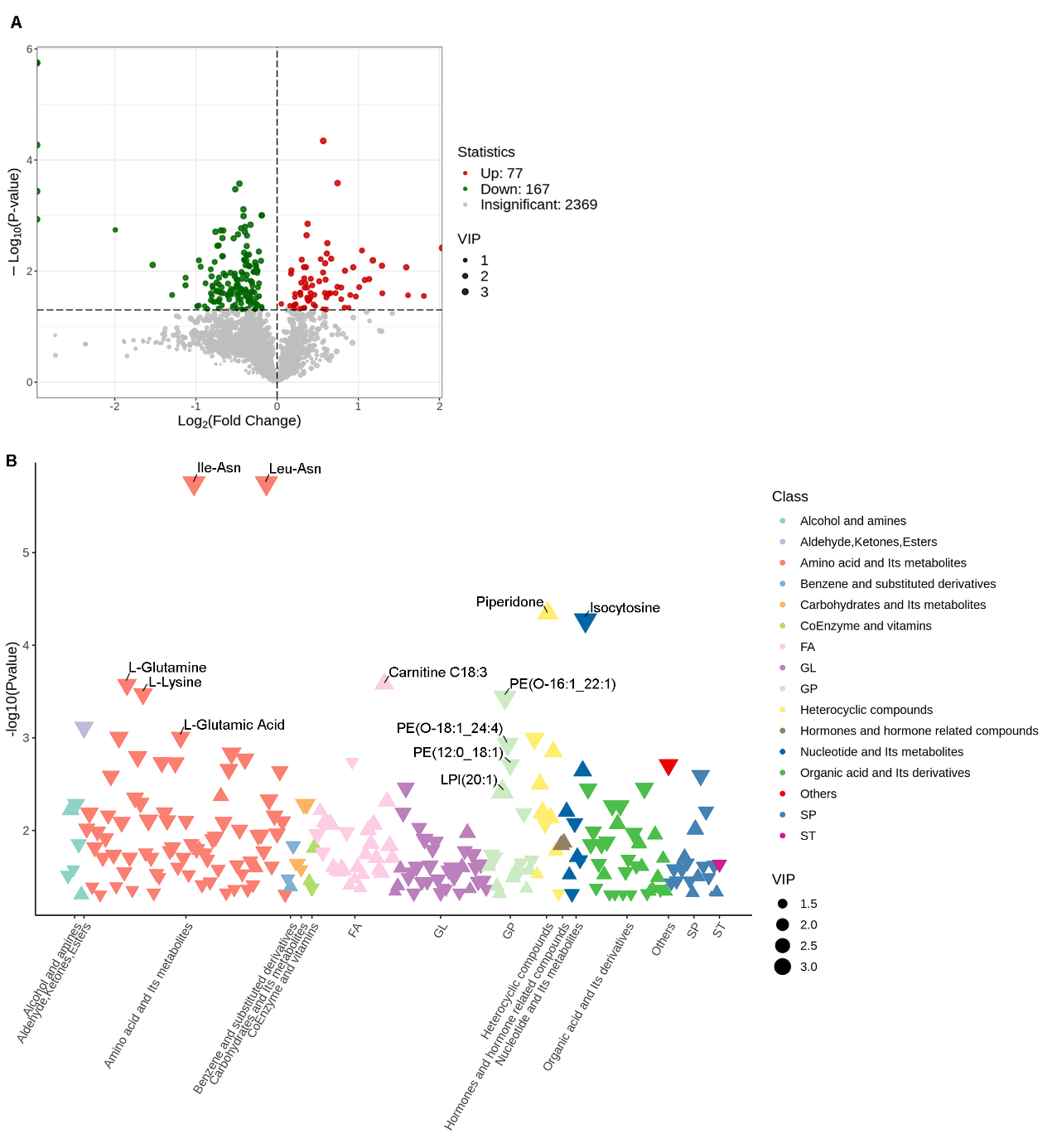


**Supplementary Figure 2: Intergroup differences in Metabolite profiles between VPA and CTRL groups revealed by principal component analysis.**

(A) The volcano plot displays the differential metabolites in VPA filtered by univariate analysis. Each point represents a metabolite: green points indicate downregulated differential metabolites, red points indicate upregulated differential metabolites, and gray points indicate metabolites detected but with insignificant differences. The x-axis represents the logarithm of the multiplicity of the differences in the relative contents of a metabolite between the two groups of samples (log_2_FC). The larger the absolute value of the horizontal coordinate, the larger the difference in the relative content of the substance between the two groups of samples.

(B) The Differential Substance Classification Scatterplot combines the results of the differential situation of substances and the category to which the substances belong, from which it is possible to visualize which types of substances are detected the most, and the difference of each type of substance in the comparison group. The horizontal coordinates of the graph below are the metabolite categories, one color represents a class of substances, and the vertical coordinates are the significance of the differences, with a -log_10_ treatment for the P value. Positive triangles represent up-regulated substances, inverted triangles represent down-regulated substances, and the size of the triangles represents the size of the VIP values.

FA, fatty acid; GL, glycerolipids; GP, glycerophospholipids; ST, sterol lipids; SP, sphingolipids.


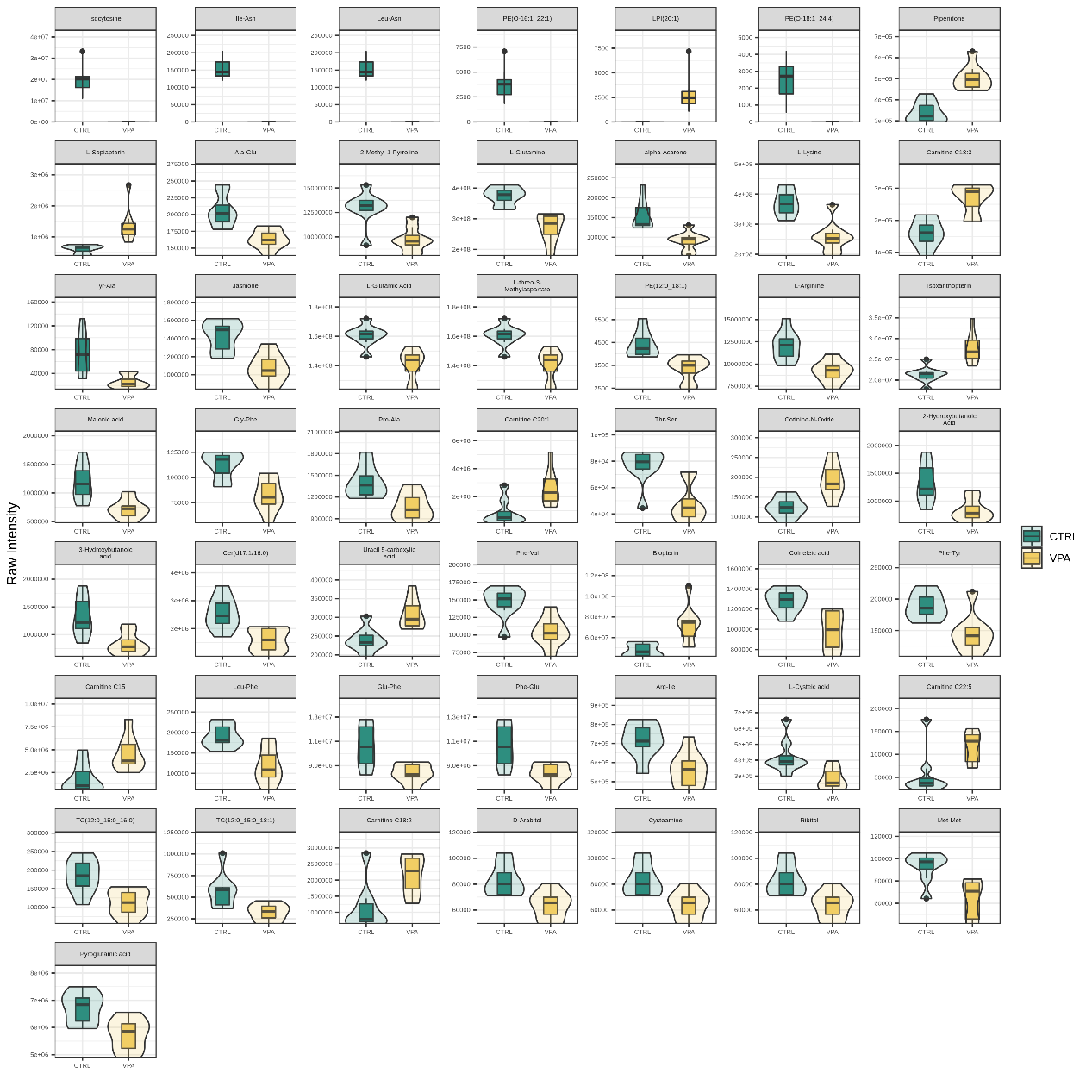


**Supplementary Figure 3: Violin plot of the top 50 metabolites with the highest VIP values.**

The horizontal coordinate is the grouping and the vertical coordinate is the relative content of the differential metabolite (raw peak area). The box shape in the center indicating the interquartile range, the thin black line extending from it representing the 95% confidence interval, and the black horizontal line right in the middle being the median, and the shape of the outer part indicating the density of the distribution of the data.

PE, phosphatidylethanolamine; LPI, lysophosphatidylinositol; Cer, ceramides.


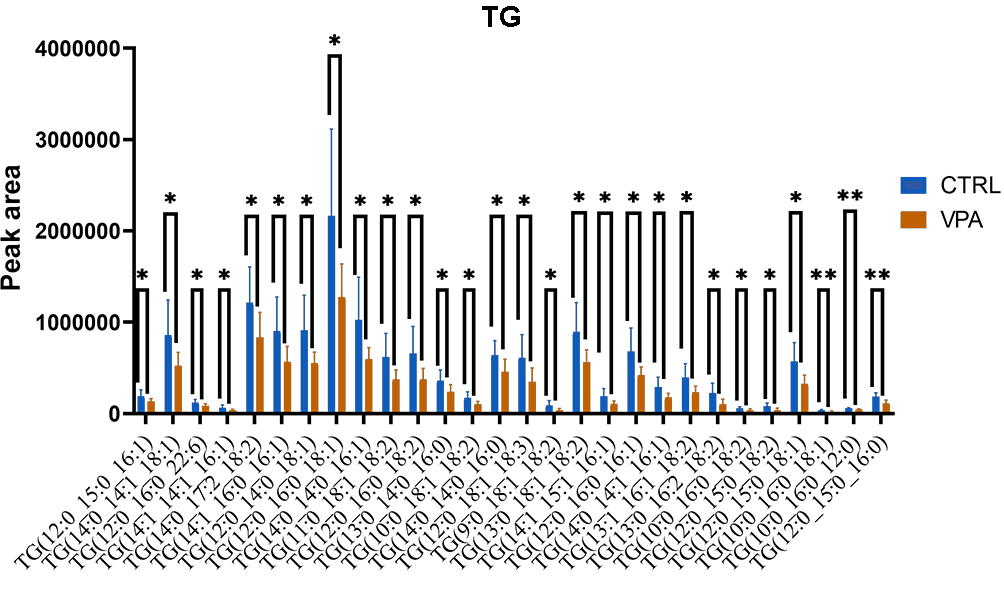
 **Supplementary Figure 4: The peak area of 28 TGs in metabolic results.**

Data are analyzed using one-way ANOVA, * *p* < 0.05, ** *p* < 0.001.
